# Pregnancy Related Health Care Needs in Refugees—A Current Three Center Experience in Europe

**DOI:** 10.3390/ijerph15091934

**Published:** 2018-09-05

**Authors:** Christian Dopfer, Annabelle Vakilzadeh, Christine Happle, Evelyn Kleinert, Frank Müller, Diana Ernst, Reinhold E. Schmidt, Georg M. N. Behrens, Sonja Merkesdal, Martin Wetzke, Alexandra Jablonka

**Affiliations:** 1 Department of Pediatric Pneumology, Allergology, and Neonatology, Hannover Medical School, 30625 Hannover, Germany; dopfer.christian@mh-hannover.de (C.D.); happle.christine@mh-hannover.de (C.H.); wetzke.martin@mh-hannover.de (M.W.); 2 German Center for Lung Research, Biomedical Research in End Stage and Obstructive Lung Disease/BREATH Hannover, 30625 Hannover, Germany; 3 Hannover Medical School, 30625 Hannover, Germany; annabelle.schaell@stud.mh-hannover.de; 4 Department of General Practice, University Medical Center Göttingen, 37073 Göttingen, Germany; evelyn.kleinert@med.uni-goettingen.de (E.K.); frank.mueller@med.uni-goettingen.de (F.M.); 5 Department of Clinical Immunology and Rheumatology, Hannover Medical School, 30625 Hannover, Germany; ernst.diana@mh-hannover.de (D.E.); schmidt.reinhold.ernst@mh-hannover.de (R.E.S.); behrens.georg@mh-hannover.de (G.M.N.B.); merkesdal.sonja@mh-hannover.de (S.M.); 6 German Center for Infection Research (DZIF), Partner Site Hannover-Braunschweig, 38124 Braunschweig, Germany

**Keywords:** pregnancy, migration, refugees, health care provision, reception center

## Abstract

*Background:* Immigration into Europe has reached an all-time high. Provision of coordinated healthcare, especially to refugee women that are at increased risk for adverse pregnancy outcomes, is a challenge for receiving health care systems. *Methods:* We assessed pregnancy rates and associated primary healthcare needs in three refugee cohorts in Northern Germany during the current crisis. *Results:* Out of *n* = 2911 refugees, 18.0% were women of reproductive age, and 9.1% of these were pregnant. Pregnancy was associated with a significant, 3.7-fold increase in primary health care utilization. Language barrier and cultural customs impeded healthcare to some refugee pregnant women. The most common complaints were demand for pregnancy checkup without specific symptoms (48.6%), followed by abdominal pain or urinary tract infections (in 11.4% of cases each). In 4.2% of pregnancies, severe complications such as syphilis or suicide attempts occurred. *Discussion:* We present data on pregnancy rates and pregnancy associated medical need in three current refugee cohorts upon arrival in Germany. Healthcare providers should be particularly aware of the requirements of pregnant migrants and should adapt primary caretaking strategies accordingly.

## 1. Introduction 

Currently, migration towards Europe is at an all-time high, and receiving countries are struggling with the task of coordinated and appropriate care provision [1]. In this situation, medical care should be adapted to the specific requirements of migrants as they represent a population with increased risk for overall morbidity and mortality [2]. This particularly holds true for pregnant women among them [3]. 

Pregnant refugee women show higher rates of adverse pregnancy outcomes, including caesarean section, stillbirth, and other maternal and perinatal morbidities [4,5,6,7,8,9,10]. 

The majority of women on the move have no access to appropriate antenatal care [11]. No or late access to antenatal care is associated with poor pregnancy outcomes [12]. Optimized maternal healthcare significantly improves pregnancy outcomes; hence, a targeted outreach to pregnant refugees may be needed to improve healthcare utilization in this patient group [3,13]. For example, refugees may carry an increased risk for intrauterinely transmitted diseases such as hepatitis, syphilis, and HIV [14,15]. Furthermore, they are at risk for insufficient vaccination against diseases such as rubella and varicella which can lead to profound and fatal outcome in their offspring [16,17]. 

Data on pregnancy associated health in the migrating population currently entering Europe is scarce. Analyzing pregnancy related health care utilization in current and representative refugee cohorts may facilitate identifying the particular needs of this vulnerable population and adapt care taking strategies accordingly. Therefore, we here analyzed pregnancy rates and healthcare utilization behavior in three representative cohorts of newly arriving refugees in Germany during the current crisis.

## 2. Methods

### 2.1. Study Population 

Data from three independent cohorts was included in the study. In total *n* = 1533 refugees residing at a reception center in Celle, Northern Germany in Summer of 2015 (from now on referred to as “cohort one”), *n* = 1220 refugees residing in 6 locations in Wolfsburg, Northern Germany in autumn 2015 (from now on referred to as “cohort two”) and *n* = 158 refugees living in a reception center in Harsefeld, Northern Germany in winter and spring 2016 (from now on referred to as “cohort three”) were included into the analysis. All three cohorts contained refugees that were allocated to a designated reception center in Lower Saxony based on a federal state-specific allocation key (Königssteiner Schlüssel). Cohorts or asylum seekers within each cohort were not preselected in any way, and data sets were chosen based on data availability and harmonization of data collection. For localization and age and gender distribution within the three reception centers, please refer to Supplementary Figure S1. Please note that part of the cohort in Celle were previously described [16,18,19]. Migrants were registered upon arrival, and their departure date was documented. For refugees leaving the center without notice to camp authorities, last contact documentation of the camp staff (medical service, food service, transportation, etc.) was used as date of departure.

### 2.2. Collection of Medical Data

A full-time medical ward offering primary medical care to all residents was erected at the center in Celle, including a medical team offering full medical services at primary care level and visiting services by a midwife. In Wolfsburg, paramedic care was offered at one of the sites and a visiting physician was available an average 2 times a week. For differences in health care utilization, only *n* = 309 refugees with on-site healthcare were included in the analysis. In the center of Harsefeld, only paramedic care was offered on site, and all other healthcare needs were referred to local physicians. For differences in health care utilization pregnant women were compared to the age matched mean of controls (refugees residing at the camp for 1 day or less were excluded from the analysis). All refugee women were asked whether they were pregnant at arrival. All refugees underwent an off-site mandatory checkup within their first weeks of residence. Prenatal care was offered to all pregnant refugees based on the standardized prenatal care guidelines [20]. All information was collected in routine clinical care. Sociodemographic information and health care data including complaints, diagnoses and prescribed medication was documented in an electronic filing system. For analysis of pregnancy associated health care utilization, the data was fully pseudonymized by the Order of Malta before scientific analysis.

### 2.3. Serological Analysis

IgG levels against varicella, measles and rubella were analyzed by Chemiluminescence Immunoassays according to the manufacturer’s recommendations (LIAISON XL, Fa. DiaSorin, Saluggia, Italy) in a diagnostic laboratory certified for routine testing (DIN ENISO 15189:2014). Threshold for protective immunity levels were: >100 IU/L for varicella (borderline 50–100 IU/L; limit of detection 10.0 IU/L), >13.4 AU/mL for measles (limit of detection 5.0 AU/mL) and >11 IE/mL (borderline 9–11 IE/mL; limit of detection 3.0 IE/mL) for rubella.

### 2.4. Statistics 

For statistical analyses, Graphpad Prism version 5.02 in combination with SPSS version 24.0 (IBM, Armonk, NY, USA) was used. To assess group differences in not normally distributed data, Mann-Whitney-U testing was applied, and *p* values below 0.05 were considered significant.

### 2.5. Ethics Compliance

All analyses were approved by local authorities (Institutional Review Board of Hannover Medical School approval # 2972-2015). All patient information was pseudonymized prior to analysis. All procedures followed were in accordance with the ethical standards of the responsible committee on human experimentation and with the Helsinki Declaration of 1964, as revised in 2013.

## 3. Results

**Figure 1:**
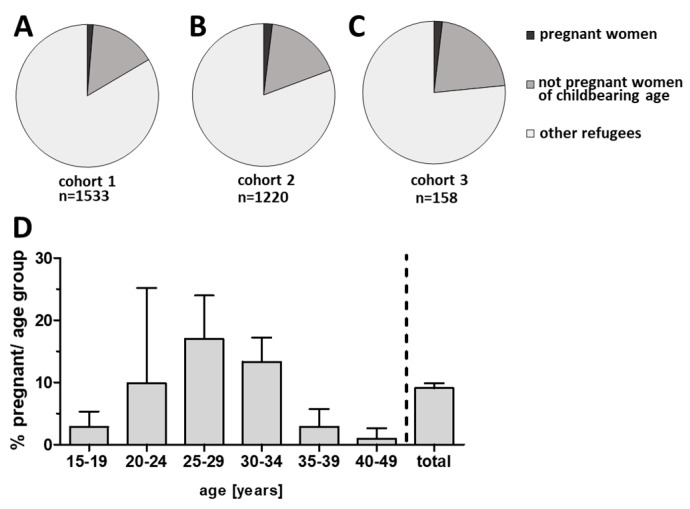
Proportion of women of childbearing age and pregnant refugees in cohort 1 (**A**) and cohort 2 (**B**), and cohort 3 (**C**). (**D**) Frequency of pregnant women among women of respective age groups and overall pregnancy rate among women of childbearing age in all three cohorts (total; bars display mean plus SD from all cohorts).

Data on pregnancy associated health care utilization in *n* = 2911 refugees from three cohorts was included into the analysis. In all three cohorts, the majority of refugees were of male gender (Supplementary Figure S1) with 71.8% of men in the largest cohort one with *n* = 1533 refugees, 65.2% of men in the large cohort two with *n* = 1220 migrants, and 63.3% of men in the smallest cohort three with *n* = 158 refugees. The proportion of women of childbearing age was 18.0% (*n* = 524; 16.3% in cohort one, 19.3% in cohort two, and 23.4% in cohort three). The frequency of women reporting to be pregnant among all refugees was 1.6% (*n* = 47), 1.3% in cohort one, 2.0% in cohort two and 1.8% in cohort three (Figure 1A–C). When we analyzed the frequency of pregnant migrants among all women of fertile age in all three cohorts (15–49 years as previously defined [21,22]), we observed a rate of 9.1 ± 0.8%, with most pregnant women in the age group 25–29 years (17.0 ± SD 7.0%, Figure 1D). Mean age of all pregnant refugees was 27.1 ± SD 5.3 years, with the youngest childbearing refugee being 16 years and the oldest one 38 years old (cohort one: mean age 27.2 ± SD 5.7 years, cohort two: mean age 25.8 ± SD 5.4 years, cohort three mean age 27.7 ± SD 3.8 years).

Most women of reproductive age (53.2%) came from Syria (61.2% in cohort one, 47.7% in cohort two and 35.1% in cohort three, Table 1). Syria was also the top country of origin of pregnant refugees (51.1%). In cohort one 60.0%, and in cohort two 45.4% of pregnant women came from this country, whereas two out of three pregnant women in cohort three came from Afghanistan and only one from Syria. Most females of reproductive age and most pregnant women were Muslims: overall, 86.1% of all females at childbearing age and 89.4% of all pregnant migrants were Muslim, 10.1% of all women at fertile age and 4.3% of pregnant females were of Christian, and 3.8% of all women at childbearing age and 6.3% of pregnant migrants in the three cohorts belonged to other religious groups or reported no belief (Table 1). Most pregnant women arrived with their husbands (85% in cohort one, 83.3% in cohort two and all pregnant women in cohort three). Overall, pregnant refugees reported no previous children in 42.6%, one in 29.8%, two in 17.0%, three in 2.1%, four in 6.4% and eight in 2.1% of cases (cohort one: 45.0% (*n* = 9) no children, 20% (*n* = 4) one, 20% (*n* = 4) two, 10% (*n* = 2) four, 5% (*n* = 1) eight children; cohort two: 33.3% (*n* = 8) no previous children, 41.7% (*n* = 10) one child, 16.7% (*n* = 4) two, 4.2% (*n* = 1) three and another 4.2% (*n* = 1) four children; cohort three: all women reported to have had no previous children, but one pregnant woman reported that she had lost one child about one year before her current pregnancy).

The majority of women of childbearing age, as well as most pregnant women, spoke Arabic, Kurdish or Persian languages (in total 60.5%, 22.2% and 19.6%, respectively, Table 2). Language barriers may have impacted the opportunities to comprehend the pregnant women’s complaints, as only 21.3% of pregnant women spoke English (15% in cohort one, 29.3% in cohort two and none in cohort three) and none reported to speak German. Lay-interpreters were available for most consultations, but not all. 35.6% of women at childbearing age in cohort one, 84% in cohort two and 97.3% in cohort three reported a profession. Of these, the occupation most reported by pregnant women as well as their non-pregnant counterparts of fertile age was housewife, followed by reported occupations as students or teachers (Table 2). 

In cohort one, *n* = 14 pregnant women reported their pregnancy during initial registration in the camp, one woman was unsure and five did not initially report or were primarily unaware of their pregnancy upon arrival at the reception center. In cohort two, one woman received test results as first confirmation of her pregnancy during camp inhabitance. In cohort three, one woman had already reported her pregnancy upon center entrance, and two early pregnancies were first detected during residence at the respective reception center. In 68.1% of pregnancies, the month of pregnancy upon first contact with the onsite medical personnel was known. Of these cases, 21.9% of women reported to be in their first, 43.8% in their second and 34.4% in their third trimester. 

**Figure 2:**
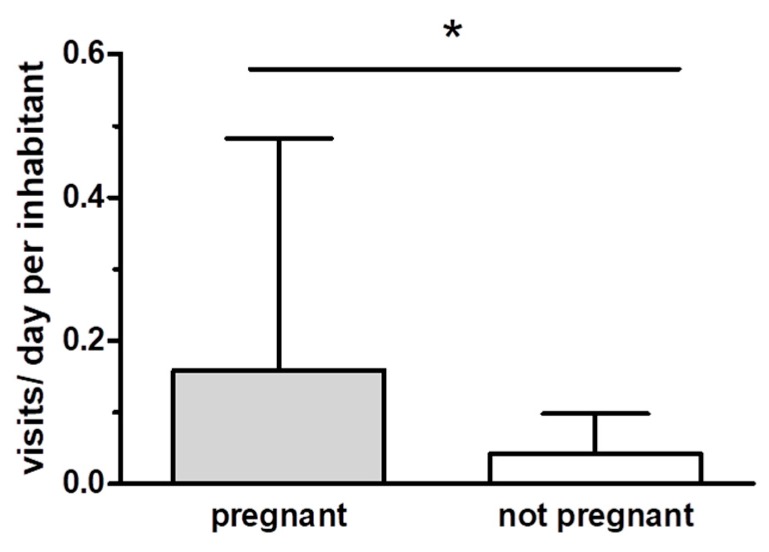
Healthcare utilization of pregnant women of the three cohorts versus the mean of the age- and gender-matched subgroup within the respective refugee cohort (bars display mean plus SD from both cohorts, * *p* < 0.05).

With regard to healthcare utilization, pregnant refugees of all three cohorts displayed a significantly higher demand for medical care compared to non-pregnant women. As shown in Figure 2, pregnant women displayed a 3.7-fold higher frequency of visits to the onsite medical unit compared to their non-pregnant female counterparts (one-tailed, *t* = 1.84, DF 52, *p* = 0.036). While pregnant women consulted the medical team at the reception center a mean of 0.16 ± SD 0.32 times per day of refugee center residence, age- and gender-matched non-pregnant women spent a mean of only 0.04 ± SD 0.05 visits to the medical unit per day of stay at the camp.

Pregnant refugees spent between 0 and 9 visits to the medical unit during a mean duration of residence of 38.8 ± SD 24.9 (mean of 2.75 ± SD 2.6 during a mean duration of stay of 31.8 ± SD 18.8 in cohort one, mean of 1.25 ± SD 1.5 during a mean duration of stay of 49.1 ± SD 39.8 in cohort two and 4.0 ± SD 2.2 during a mean duration of stay of 78.3 ± 17.1 SD in cohort three). When analyzing all visits to the onsite medical units in both camps, the reason of consultation was reported in 96.8% of visits. The most frequent demand in all consultations of pregnant refugees was to receive a general checkup by a specialized obstetrician or midwife without specific complaints (48.6% of consultations). The most frequent specific symptoms or diagnoses pregnant women presented with were abdominal pain (11.4%) or urinary tract infections (11.4%), followed by symptoms such as skin rash and itching (8.6%). Overall, 54.4% of consultations were because of general pregnancy-related medical demands for checkups or nutritional supplements, 25.7% because of pain-related problems, 20% because of infections, and another 20% because of other, less frequent complaints or diagnoses (Table 3). No woman asked for abortion. 

Out of all pregnant migrants in the three cohorts, in two pregnant women (4.2% of all pregnancies), severe complications were diagnosed, necessitating immediate expert care: one pregnant migrant in cohort one had a positive syphilis serology during routine testing and was treated with antibiotics. In cohort three, one depressed refugee attempted to commit suicide during her second month of pregnancy and was admitted to the hospital for five days. After hospitalization, this woman was closely monitored with weekly gynecologist checkups and psychological support until she moved out of the reception center. 

In nine pregnant women from cohort one, information on serological screening for immunoglobulins (Ig) against infectious diseases was available. One woman tested positive for anti-hepatitis B core antigen, as well as anti-hepatitis B surface antibodies, but negative for hepatitis B surface antigen. None of the pregnant refugees had positive screening results for hepatitis C, D, or E. All tested pregnant migrants were seropositive for IgG against varicella, and 89% of them were seropositive for measles-IgG. However, with regard to anti-rubella IgG, only 44% of pregnant refugees had protective titers, and an additional 22% had borderline protective levels of IgG against this vaccine preventable disease. 

The onsite medical ward in cohort one, which offered daily physician attendance and regular midwife consultations, provided medical care for most of the problems that occurred during consultations. Cohort two had regular access to an attending physician (average 2 times a week) and cohort three had unrestricted access to a close by general practitioner that provided medical care for most of the acute problems. However, thorough obstetric checkups that were requested by 48.6% of pregnant women, as well as the suicidal female refugee had to be referred to specialized physicians as their problems exceeded the level of primary onsite care. Of note, one pregnant Muslim wanted to only be examined by female doctors and preferred not to be treated over being examined by a male physician, who was the only available doctor at the onsite ward on that day. 

## 4. Discussion

Immigration into Europe has reached an all-time high, and provision of coordinated healthcare poses an enormous challenge for receiving communities [1,23,24]. Medical care is key in management during humanitarian crises as the current, and especially for refugee woman, that are at increased risk for adverse pregnancy outcomes, caretaking strategies need to be adapted [3,25,26]. Accordingly, we analyzed pregnancy rates and pregnancy-associated primary healthcare utilization in three representative cohorts of newly arriving migrants in Western Europe. 

In total, healthcare utilization data of *n* = 2911 refugees from three cohorts was included into the analysis. Both cohorts contained large proportions of young adult males and the majority of refugees came from the Eastern Mediterranean region, both typical demographic characteristics of current European immigration statistics [17,27,28,29]. In these representative cohorts, 18% of refugees were females of fertile age, and 9.1% of these women were pregnant. These pregnancy rates are comparable with previous publications. While it is challenging to obtain accurate statistics on the exact frequency of pregnancies among female migrants, the women’s refugee commission reports that at any given time 0.6 to 14 percent of all displaced women between 15 and 49 years could be pregnant, and other authors estimate that, depending on country of origin, even higher proportions of up to 25% of female refugees of fertile age could be pregnant [22,26,30]. 

Refugees are at particular risk for infectious diseases, for physical and psychological trauma, sexual violence and for insufficient access to healthcare and prevention programs, as well as contraception [1,11,17,31]. This particularly holds true for pregnant migrants [3,25,26]. Up to 15% of women who are pregnant while fleeing their homelands experience life-threatening obstetric complications, and Simsek et al. recently reported a frequency as high as 47.7% of pregnancy losses among Syrian refugee women living in Turkey [22,26]. Multiple studies have confirmed increased rates of adverse pregnancy outcomes in migrants, including reduced fetal growth, caesarean section, stillbirth, maternal depression and other maternal and perinatal morbidities [4,5,6,7,8,9,10]. 

Consequently, pregnant refugees should receive particular medical attention, especially when arriving in a country with high economic and healthcare standards such as Germany. Indeed, in our observations of newly arriving refugees, pregnancy was associated with a significant, 3.7-fold increase in primary health care utilization. The most common reasons for medical consultations by pregnant refugees in our cohorts were the demand for pregnancy checkups or prescription of nutritional supplements without acute symptoms, followed by abdominal pain or other pain related issues and less frequent problems such as headache or infections. 

One pregnant woman in our cohorts tested positive for hepatitis B core antigen without presence hepatitis B surface antigen. Another pregnant refugee suffered from syphilis and was treated immediately to prevent vertical transmission. Even though, in the first case, test results suggested no immediate threat to the offspring, and treatment was successful in the second case, both observations illustrate the importance infectious disease screening in migrants, especially in pregnant refugees, as the prevalence of severe, vertically transmittable diseases is higher in migrants than in the general population [14,32]. Also, we observed an alarmingly low rate of anti-rubella seropositivity in the small specimen of serologically tested pregnant refugees: only 44% of the expecting mothers had protective anti-rubella IgG-levels. Even though seroprevalences do not necessarily reflect immunity acquired by vaccination, this observation is in line with previous reports by us and others and illustrates a significant gap in rubella immunity in young female refugees [16,17,33,34,35].

One woman within our cohorts attempted to commit suicide while being pregnant. Although this is just a single observation, it is in line with the results of multiple observational studies reporting on the increased burden of mental diseases and depression in refugee women during pregnancy and the perinatal phase [36,37,38]. Factors such as social isolation, poverty, lack of host language skills and belonging to an ethnic minority have been described to put pregnant refugees at increased risk of mental disorders [37]. Especially for pregnant women prone to depression, access to psychological help and appropriate support programs should be facilitated, as at least preliminary data shows that the latter measure reduces the rate of mental disorders in Syrian refugee mothers arriving in Canada [26].

Pregnancy outcomes in migrants are influenced by several factors such as country of origin, race and destination country [39]. During their migration, pregnant women only rarely have access to appropriate health care services along the way [22]. Healthcare provision in the receiving country is a main factor in maternal health. For example, Syrian refugees in Jordan experience significantly higher rates of perinatal complications, including iron deficiency, caesarian section, and low birth weight than Jordanian women, but Turkish and Syrian refugee women in Turkey have been reported to show similar pregnancy outcomes [40,41,42]. In Lebanon, the United Nations High Commissioner for Refugees covers 75% of the cost of life-saving, obstetric, and emergency hospital care for migrants, but the remaining 25% is oftentimes unaffordable for refugees, leading to high morbidity and mortality, particularly in pregnant migrants [43].

Besides structural and organizational barriers, social, personal and cultural factors may significantly impact healthcare utilization in pregnant migrants. Language barriers and cultural customs can significantly impede healthcare to some pregnant refugee women. For example, only the minority of pregnant women in the here-analyzed cohorts spoke English, and none of them spoke the host language, German; thus, without interpreters, medical problems could not be fully communicated between patient and doctor. Furthermore, cultural customs may have impacted healthcare utilization in our observation. In both cohorts, most of the pregnant women were Muslims, and one of them refused to be examined by a male doctor. Cultural background has been previously described to significantly impact peripartum care and well-being in refugee women. In general, medical staff taking care of newly arriving refugees should consider the probability of a limited understanding of Western medicine in refugee women, and avoid them feeling forced to adapt, being labelled as non-compliant if they resist Western approaches [3]. Our experience supports the notion that appropriate language interpretation and the availability of female medical staff could facilitate healthcare utilization for pregnant refugees.

Our study has important limitations. Although the demographics of our cohorts mirror current migration statistics, they can only represent a small specimen of refugees entering Europe during the current crisis. Especially in the smaller cohort and in specific demographic subgroups of the larger cohort, the low number of subjects needs to be taken into account when interpreting our data. Another limitation may lie in the fact that we could only analyze self-reported pregnancies and, due to the fact that our data collection was conducted during routine clinical care, it could not be controlled for language or cultural barriers that may have impacted the refugees answer to the question of pregnancy upon entrance into the reception center or at health care encounters. The same limitation may also have impacted the documentation of complaints. Furthermore, we were unfortunately unable to follow-up on pregnancy outcomes, as all women were moved to their permanent location of residence after registration by the German asylum agency.

## 5. Conclusions

Optimized maternal healthcare is an effective method to improve pregnancy outcomes as well as lifelong maternal and offspring health, and a targeted outreach to pregnant refugees may be needed to improve utilization of beneficial care [3,13]. 

The here presented data may facilitate the setup of an appropriate outreach of this kind. It confirms that pregnant migrants are a patient group with increased healthcare utilization and particular medical needs. Primary care providers offering medical help during the current crisis should be aware of the high demand for obstetric checkups in pregnant migrants and ideally be supported by interpreters capable of speaking Arabic and Persian languages. Furthermore, they should consider religious and cultural customs of arriving pregnant migrants, for example female staff could be preferred over male doctors offering obstetric care. Moreover, an increased burden of psychological stress during escape should be considered in pregnant women compared to their non-pregnant counterparts. Also, it should be kept in mind that effective reproductive healthcare starts well before pregnancy, when preventive measures such as screening for infectious diseases, rubella vaccination, or alimentary supplementation need to be commenced. 

We hope that our data on the particular healthcare demands of pregnant refugees may help to adapt care-taking strategies in this particularly vulnerable patient group.

## Figures and Tables

**Table 1:** Cohort-specific characteristics of women of childbearing age and pregnant refugees, country of origin and religion.

	Total		Cohort 1		Cohort 2		Cohort 3	
	Women of Child-Bearing Age *n* = 524	Pregnant Women *n* = 47	Women of Child-Bearing Age *n* = 250	Pregnant Women *n* = 20	Women of Child-Bearing Age *n* = 237	Pregnant Women *n* = 24	Women of Child-Bearing Age *n* = 37	Pregnant Women *n* = 3
country of origin (Top 10)	%	%	%	%	%	%	%	%
Syria	53.2	51.1	61.2	60.0	47.7	45.4	35.1	33.3
Afghanistan	17.7	21.3	7.2	5.0	26.6	27.3	29.7	66.6
Iraq	13.5	14.9	6.4	5.0	20.3	27.3	18.9	0
Iran	3.4	0	1.6	0	3.8	0	13.5	0
Eritrea	2.5	0	4.8	0	0	0	2.6	0
Albania	1.9	2.1	4.0	5.0	0	0	0	0
Serbia	1.1	0	2.8	0	0	0	0	0
Azerbaijan	1.0	4.1	2.0	10.0	0	0	0	0
Bosnia	0.4	2.1	0.8	5.0	0	0	0	0
Montenegro	0.4	2.1	0.8	5.0	0	0	0	0
Nigeria	0.2	2.1	0.4	5.0	0	0	0	0
religion								
Muslim	86.1	89.4	85.6	90.0	88.6	91.7	73.0	66.7
Christian	10.1	4.3	10.8	5.0	8	0.0	18.9	33.3
Others/unknown	3.8	6.3	3.6	5.0	3.4	8.3	8.1	0

**Table 2:** Cohort-specific characteristics of women of childbearing age and pregnant refugees, language skills and profession.

	Total		Cohort 1		Cohort 2		Cohort 3	
	Women of Child-Bearing Age *n* = 524	Pregnant Women *n* = 47	Women of Child-Bearing Age *n* = 250	Pregnant Women *n* = 20	Women of Child-Bearing Age *n* = 237	Pregnant Women *n* = 24	Women of Child-Bearing Age *n* = 37	Pregnant Women *n* = 3
Languages (Top 5)	%	%	%	%	%	%	%	%
Arabic	60.5	59.6	62.4	55.0	61.1	66.7	43.2	33.3
English	16.1	21.3	20.4	15.2	13.2	29.3	5.4	0
Kurdish languages	22.2	14.9	16.4	10.0	26.7	20.9	32.4	0
Persian languages	19.6	10.0	8.8	5.0	27.4	7.0	43.2	66.6
Albanian	1.9	2.1	4.0	5.0	0	0	0	0
Profession (top 5)								0
None/unknown	38.2	36.2	64.4	65.0	16.0	16.7	2.7	0
Housewife	29.9	34.1	9.6	20.0	46.7	41.7	59.5	66.6
Student	13.8	8.5	8.8	5.0	19	12.5	13.	0
Teacher	5.1	4.3	4.0	0	5.8	4.2	8.1	33.3
Tailor	1.9	2.1	2.0	0	1.7	4.2	2.7	0
IT specialist	0.4	2.1	0.8	5.0	0	0	0	0
Hairdresser	1.7	4.3	0.8	5.0	3	4.2	0	0

**Table 3:** Overview on pregnancy associated medical complaints and requests (proportion of all consultations in all three cohorts).

Complaints	% of Cases	Complaints	% of Cases
pregnancy related. no acute complaints	54.3	infections	20.0
demand for obstetric checkup	48.6	urinary tract infection	11.4
demand for pregnancy supplements	5.7	syphilis	2.9
iron deficiency	iron deficiency	respiratory infection	5.7
pain	25.7	others	20.0
abdominal pain	11.4	skin rash/ pruritus	8.6
toothache	2.9	hyperventilation	2.9
backpain	2.9	dyspnea	2.9
headache	5.7	weakness	2.9
physical trauma	2.9	depression. suicide attempt	2.9

## Supplementary Materials

The following are available online at http://www.mdpi.com/1660-4601/15/9/1934/s1.
