# Supplementary material for: Pregnancy Related Health Care Needs in Refugees—A Current Three Center Experience in Europe
Source: Int J Environ Res Public Health. 2018 Sep 5;15(9):1934. doi: 10.3390/ijerph15091934 (PMC6165089; doi:10.3390/ijerph15091934)
Supplement: Supplementary file 1 [file ijerph-15-01934-s001.pdf]

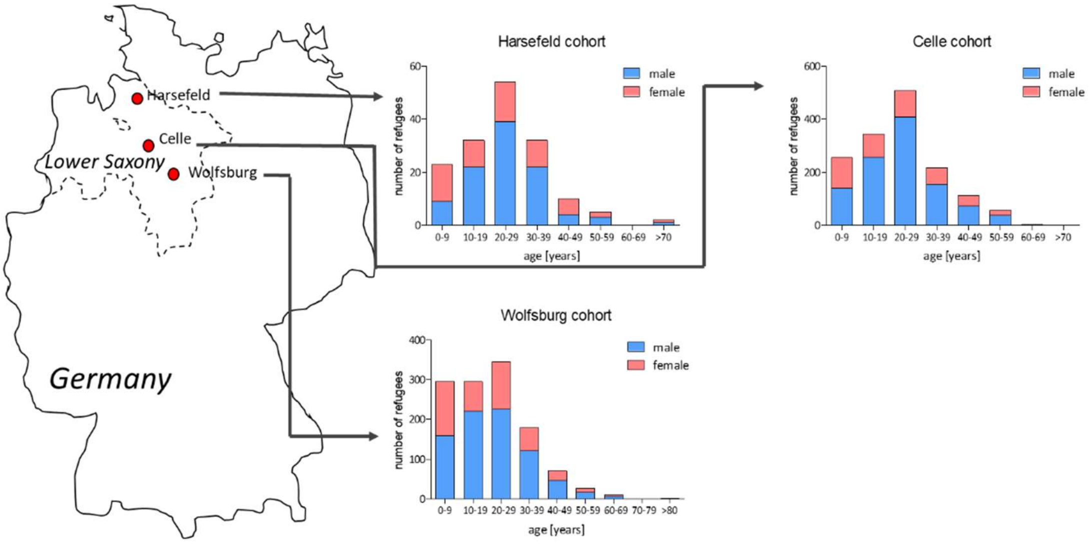

Figure S1. Localization of reception centers and age and gender distribution in the three described cohorts. Note that part of the cohort in Celle were previously described (16, 18, 19).
